# Unveiling an Uncommon Scenario of Co-occurrence of Multiple Odontomes With Impacted Maxillary Lateral Incisor and Canine in a 17-Year-Old Girl: A Unique and Rare Case Report

**DOI:** 10.7759/cureus.61435

**Published:** 2024-05-31

**Authors:** Hitesh Sawant, Parag V Gangurde, Sneha Padmakarrao Masne, Sheetal M Jadhav, Amit Patil, Shreyas Shah, Sayem A Mulla, Saba Kondkari

**Affiliations:** 1 Department of Orthodontics and Dentofacial Orthopedics, Bharati Vidyapeeth Dental College and Hospital, Navi Mumbai, IND; 2 Department of Oral Pathology and Microbiology, Bharati Vidyapeeth Dental College and Hospital, Navi Mumbai, IND; 3 Department of Prosthodontics, Bharati Vidyapeeth Dental College and Hospital, Navi Mumbai, IND; 4 Department of Conservative Dentistry and Endodontics, Bharati Vidyapeeth Dental College and Hospital, Navi Mumbai, IND; 5 Department of Oral Medicine and Radiology, Bharati Vidyapeeth Dental College and Hospital, Navi Mumbai, IND; 6 Department of Dentistry, Bharati Vidyapeeth Dental College and Hospital, Navi Mumbai, IND

**Keywords:** overretained deciduous teeth, impacted teeth, regional accelerated phenomenon, absolute anchorage, odontome

## Abstract

This case report presents the enigma of multiple odontomes with overretained deciduous teeth leading to the impaction of permanent successors (22, 23) in an abnormal position in a 17-year-old female patient who reported the chief complaint of maligned teeth. Permanent maxillary canines and lateral incisors are the most common teeth to face the brunt of impaction due to a wide range of etiological factors. It is imperative for a clinician to diagnose cases at an early stage to accelerate the rate of eruption of such teeth. This is especially important in cases where initially the etiology seems to be simple but on careful and judicious evaluation of the case, numerous other etiologies are found to map together for the underlying pathology. This case discusses how the presence of multiple odontomes with delayed exfoliation of deciduous teeth leads to the impaction of a permanent successor. Understanding the underlying pathology is seemingly important to devise intricate treatment mechanics for traction of impacted teeth without taxing anchorage from dental units and taking cognizance of the amount of alveolar bone loss post-removal of multiple odontomes. The appropriate thickness of alveolar bone scaffolding is required for the canine to extrude down, with an adequate band of marginal gingiva encircling the cement-enamel junction of the impacted canine, preventing any kind of fenestration and dehiscence. Hence, meticulous care was taken during surgical exposure and removal of odontomes to preserve an adequate labial cortical plate intact for traction. These excavated tooth-like structures were later subjected to histopathological evaluation, which confirmed the diagnosis of compound odontomes.

## Introduction

Maxillary canines being the cornerstone of the arch play an important role in function and aesthetics. Interestingly, canine impaction is more than twice as common in the maxilla compared to the mandible [1]. Overretained deciduous teeth impede the path of eruption of permanent teeth, leading to various types of malocclusions. The autonomous eruption of a permanent successor after the removal of overretained deciduous teeth depends on its proximity to the root of fully erupted adjacent teeth in the arch. Horizontal overlap of the incisor root governs the path of eruption of the impacted canine following the removal of overretained deciduous teeth [1]. The maxillary canine has a tortuous path of eruption in the oral cavity, while the root of the maxillary lateral incisor serves as a guide for the eruption of permanent canine [2]. Maxillary canine impaction is commonly encountered when overretained deciduous teeth block its path of eruption. In the aforementioned case, impaction of the maxillary lateral incisor further impeded the path of eruption of the permanent canine. Hamartomas of interrupted tooth-formed structures are basically odontomas, which account for 22% of the odontogenic tumors [3]. The overlying odontomes in the region of 22-23 further accentuated the complexity of the scenario. 

The co-occurrence of multiple pathologies together often puts the operator in a diagnostic and therapeutic dilemma. The current case presents one such unique case, representing a clinico-therapeutic conundrum of the co-occurrence of multiple impacted teeth with supernumerary odontomes.

## Case presentation

**Figure 1:**
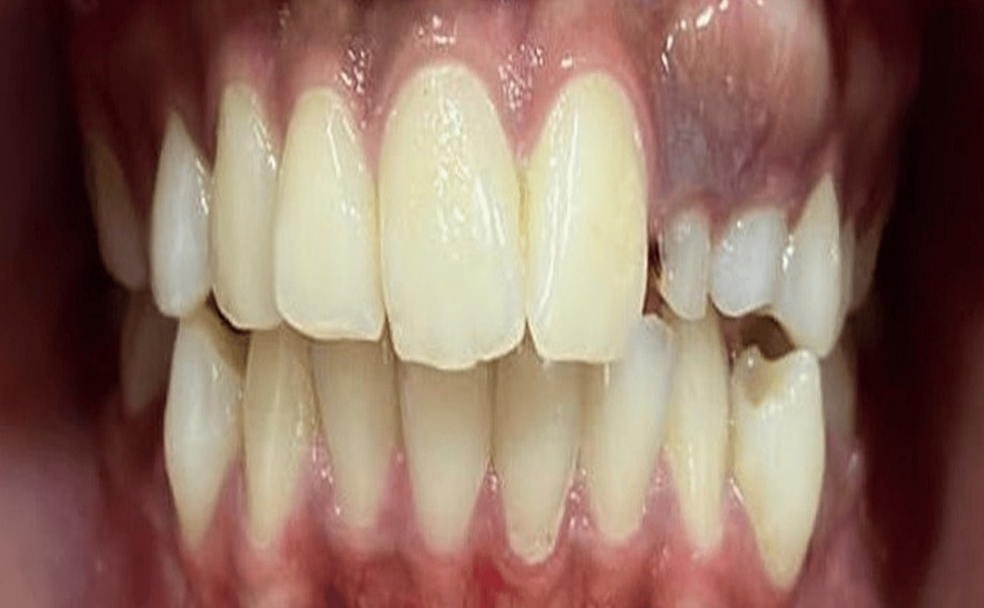
Pretreatment frontal photographs depicting a bony hard bulge in the maxillary anterior region

A 17-year-old girl reported a chief complaint of painless swelling in the upper anterior esthetic tooth region (Figure 1).

**Figure 2:**
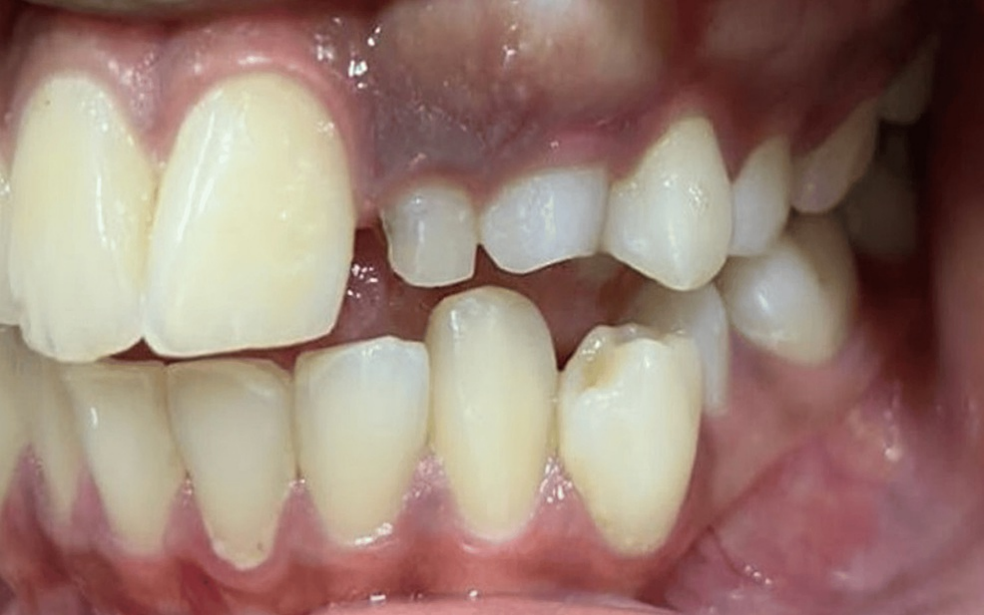
Pretreatment intraoral view of dentition depicting bony hard bulge distal to 21 and mesial to 24

On intraoral examination, there was the presence of overretained deciduous 62 and 63 and no signs of autonomous eruption of 22 and 23 to be expected (Figure 2).

**Figure 3:**
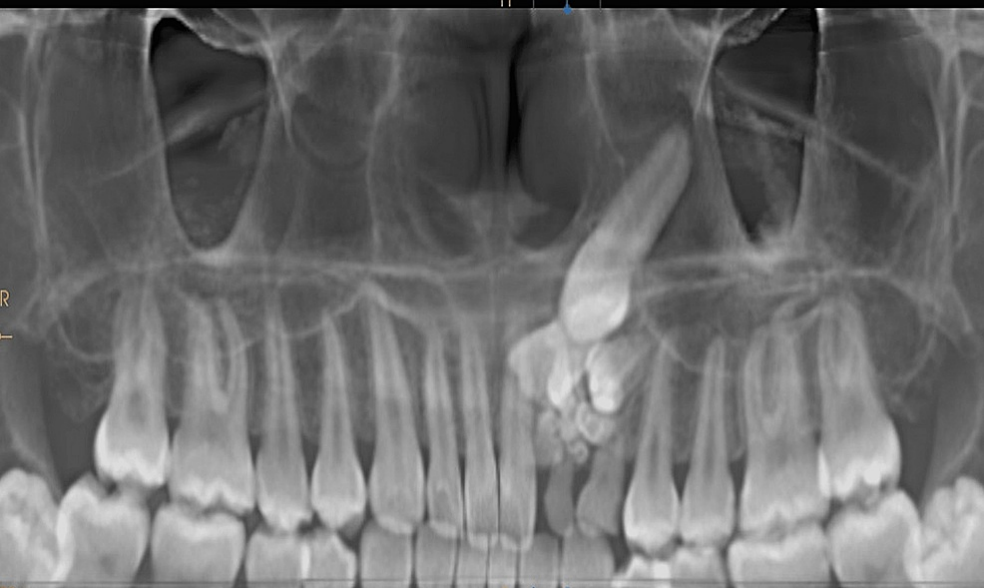
OPG showing overretained 62 and 63 with radioopaque tooth-like structures overlying the deciduous teeth, with mesiolabially tipped 23 at the level of root apices of 21 and 24 OPG, orthopantomograph

The patient presented with bony-hard swelling on the alveolar mucosa of the upper left quadrant, specifically in relation to the 62 and 63 regions, with the absence of a draining sinus. The presence of swelling in this area is suggestive of either an odontogenic infection, inflammation, or even a cyst. An orthopantomograph (OPG) revealed numerous radio-opaque structures in the 62-63 region, along with the presence of impacted 22 and 23 (Figure 3).

**Figure 4:**
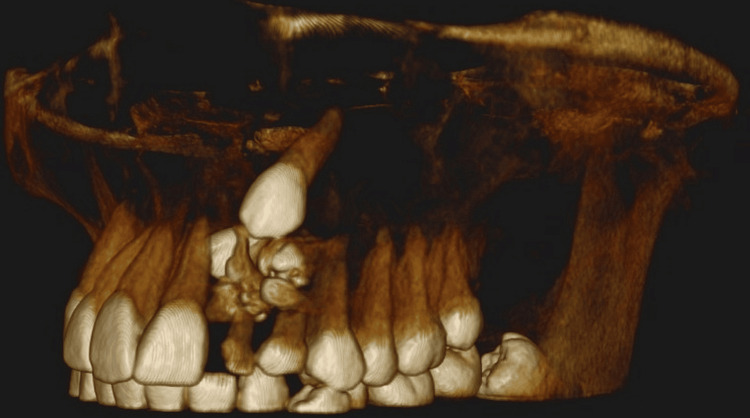
CBCT image of impacted canine with odontome incisal to cusp tip of 23 and thinning of the labial cortical plate CBCT, cone beam computed tomography

Given these findings, the patient was advised to undergo a cone beam computed tomography (CBCT) scan, which revealed multiple radiopaque tooth-like structures approximately 10-12 in number along with an impacted 22 and 23 with a thin buccal cortical plate along the crown of 22 (Figure 4).

These findings provided crucial information for the treatment plan. The presence of multiple radiopaque tooth-like structures required further histopathological investigation to determine their nature and any associated risks. Overall, histopathological analysis provides valuable insights into the nature and potential risks of the radiopaque structures, enabling the dental team to make informed decisions regarding the patient's care.

**Figure 5:**
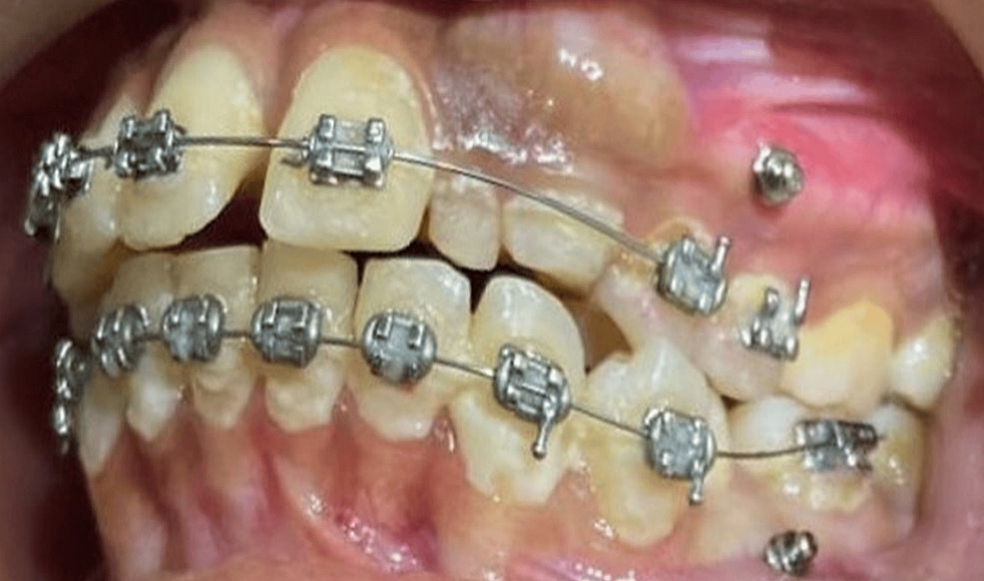
Mini implant placement for traction of impacted canine

Taking cognizance of the complexity of the clinical scenario, a treatment plan was devised for traction of impacted teeth without anchorage of adjacent permanent teeth in an attempt to prevent inadvertent tooth movement. 1.6*8 mm mini implants (TADs) were placed in the maxillary posterior region under 2% Lignox (lignocaine with adrenaline) (Figure 5).

**Figure 6:**
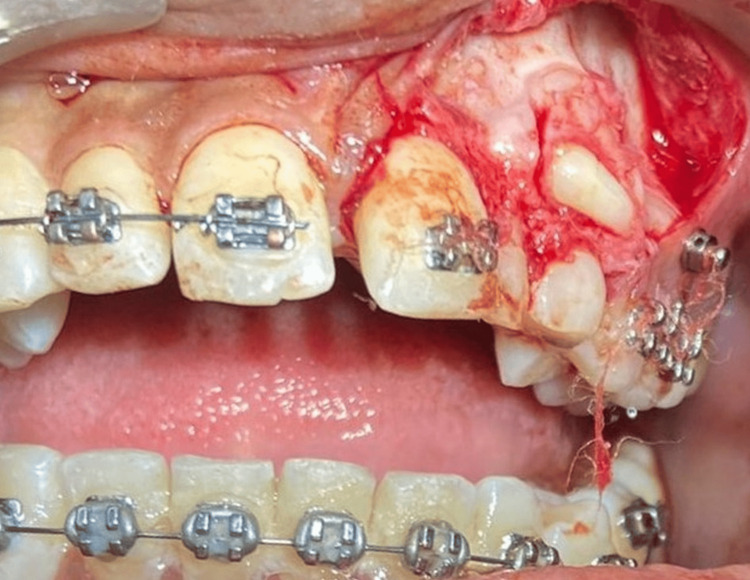
Surgical exposure of the site with an overlying odotome resembling the tooth-like structure

**Figure 7:**
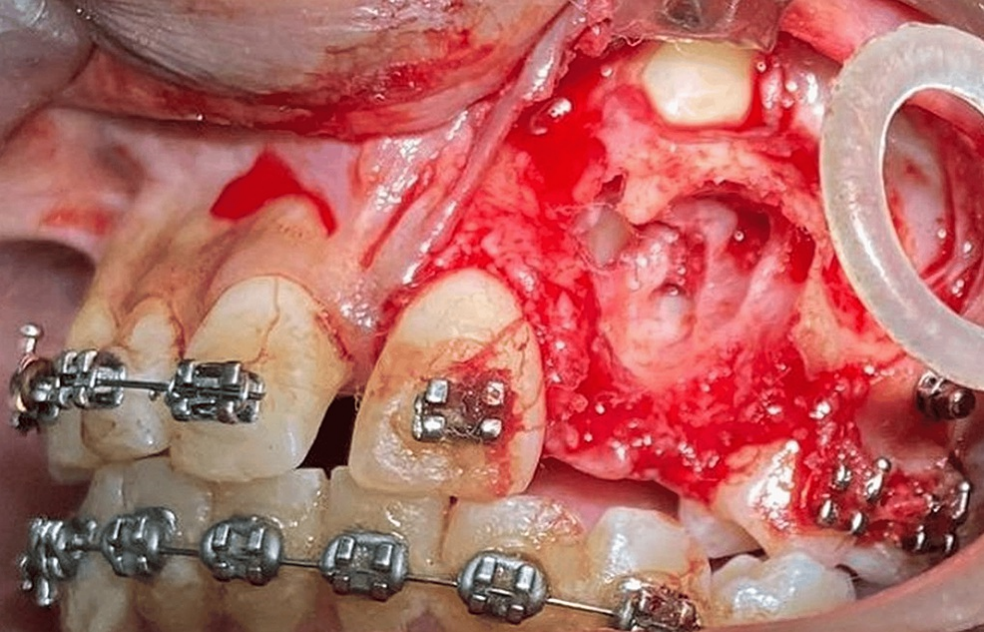
Removal of odontomes and exposure of the facial surface of canine to facilitate placement of the attachment

A full-thickness flap was raised and reflected with a periosteal elevator. The overretained deciduous lateral incisor and canine were extracted. Careful removal of odontomes was planned to create a site for the bonding of attachments on impacted teeth (Figures 6, 7).

**Figure 8:**
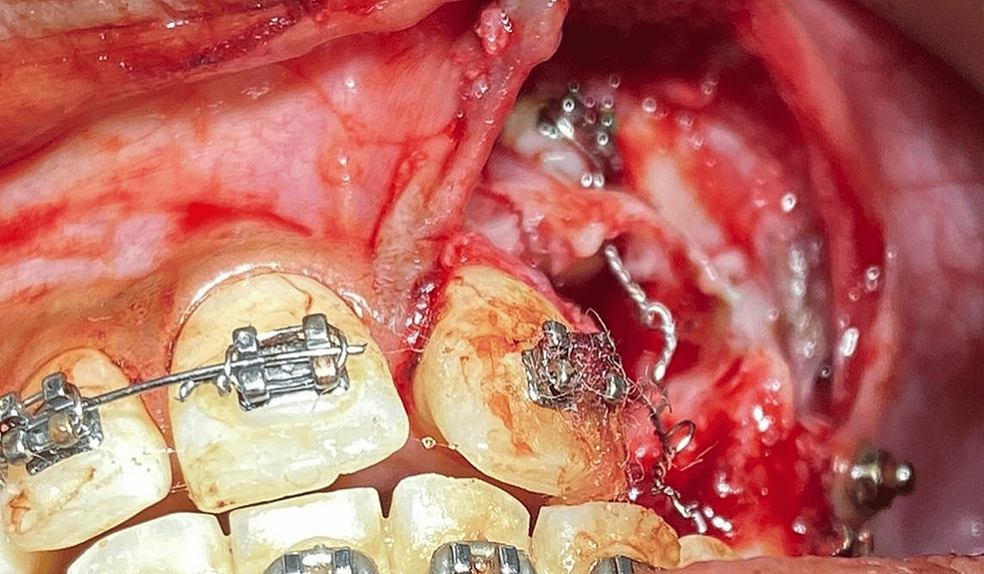
Attachment with eyelet bonded to the accessible facial surface of maxillary impacted canine

After the removal of odontomes, a moisture-insensitive primer was placed to initiate efficient bond strength between the bonded attachment and tooth surface in the blood-contaminated field. Attachment with an eyelet type of design was placed with an additional ligature type J hook placed in the eyelet to aid in the traction of impacted teeth (Figure 8).

## Discussion

An odontoma is a benign tumor of odontogenic origin that is formed from both the epithelial and mesenchymal components of the odontogenic apparatus. The infiltration of extra-odontogenic epithelial cells from the dental lamina leads to the development of an odontoma. The odontoma progresses through the same stages as the developing tooth. Initially, there is resorption, and the lesion is radiolucent. In the intermediate stage, the odontogenic tissue becomes radiolucent and radiopaque due to partial calcification. The most radiopaque stage occurs when the calcification of dental tissues is complete [4].

In the present case, there was the occurrence of multiple (10 individual) odontomes in the lateral incisor-canine region (Figures 3, 4), suggesting that it was a compound odontome, which has an affinity for being in the maxillary anterior segment, i.e., about a 62% occurrence rate is seen [5]. In the case of a missing permanent lateral incisor, the permanent maxillary canine finds it difficult to erupt as the roots of the laterals serve as guidance for its eruption. The occurrence of these multiple odontomes in the path of eruption of the 22 and 23 could also be a possible reason behind the impaction of these teeth in the present case.

Extraction of overretained deciduous lateral and canine was imperative to create a path of eruption for succedaneous blocks in permanent lateral and canine. The anchorage requirement is critical for the traction of impacted canines. If adjacent permanent teeth bonded with a preadjusted edgewise appliance are utilized for traction of impacted teeth using the piggyback technique, it ushers in the need for full-thickness wire in the bracket slot to prevent inadvertent tooth movements during the extrusion of impacted canines. It takes seven to eight months, at times, from the inception of fixed orthodontic treatment to reach the full-thickness archwire. In an attempt to reduce this time frame and initiate the extrusion of impacted teeth at the outset, it was pivotal to incorporate strong anchorage control units into the treatment plan. Temporary anchorage devices (TADs) were decided to be used for traction of impacted permanent teeth, as this would not tax the anchorage. The use of TADs for traction of impacted permanent teeth during fixed orthodontic mechanotherapy helped reduce taxation on dental units. Also, patients' burnout phase is eliminated or significantly reduced as traction is initiated at the outset. Immediate traction of impacted teeth helps to increase the pace of tooth movement, as extraction of over-retained teeth followed by removal of odontomes simulates an environment similar to RAP [6-8].

**Figure 9:**
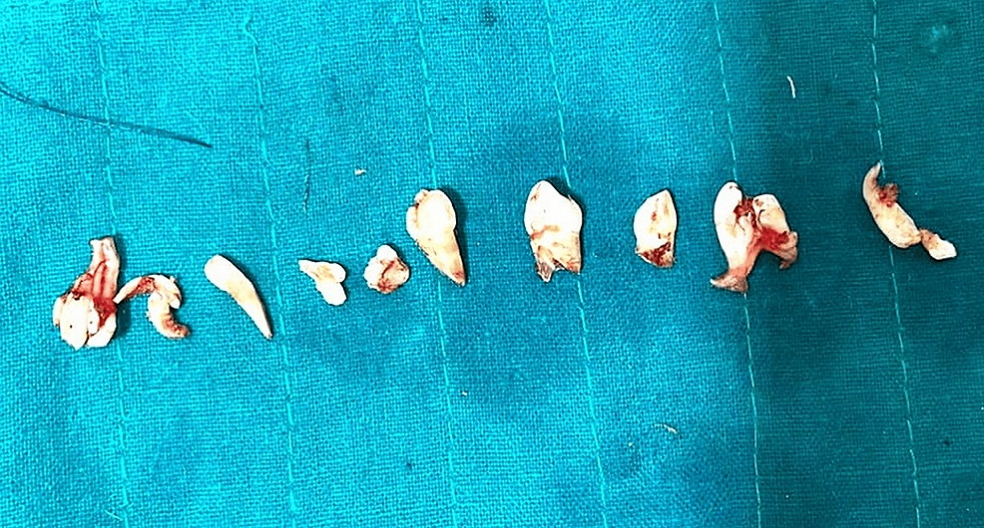
Macroscopic aspects of 10 tooth-like structures that have been surgically removed

An odontoma, being the most common odontogenic tumor, is frequently associated with the development of a calcifying cystic odontogenic tumor (CCOT) in 24% of cases [9,10]. The elimination of these odontomes will help prevent any future risk of the development of a cyst. Post-operatively, 10 tooth-like structures were obtained (Figure 9).

**Figure 10:**
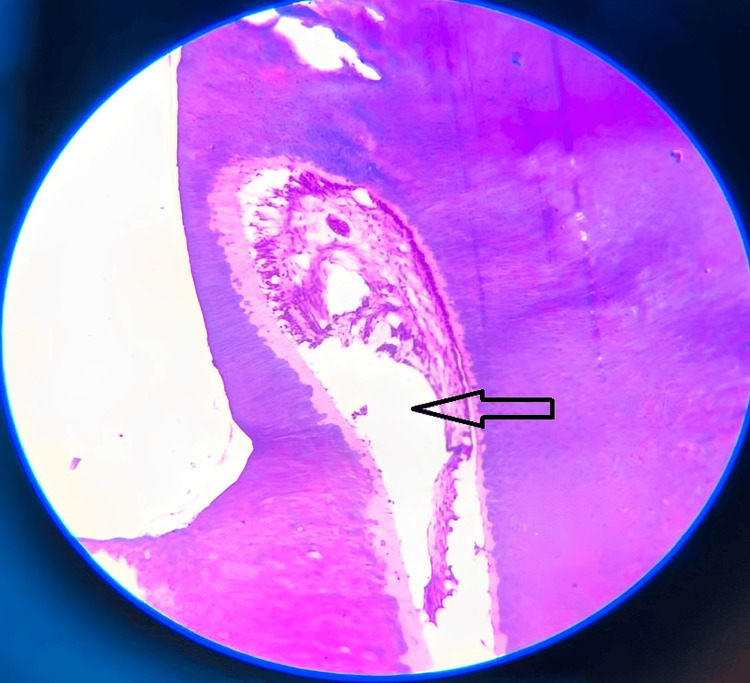
The photomicrograph shows enamel space and the longitudinal section displays dentinal tubules and inner delicate connective tissue with few blood vessels and inflammatory cells resembling pulp tissues The arrow shows enamel space

These structures were then subjected to decalcification using 5% nitric acid. The decalcified H and E stained tissue sections of multiple bits of the specimen collected revealed enamel space, a longitudinal section of dentinal tubules, and inner delicate connective tissue with few blood vessels and inflammatory cells resembling pulp tissue (Figure 10).

**Figure 11:**
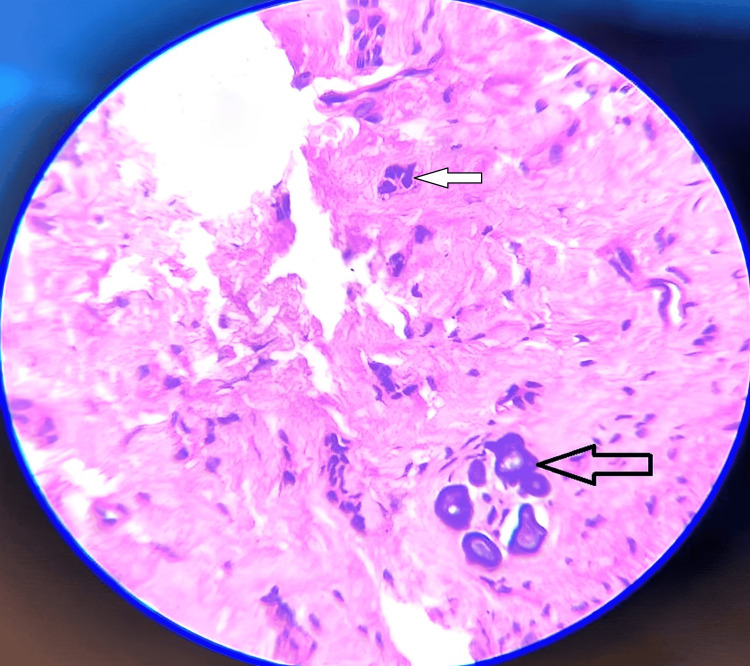
Photomicrograph of H and E decalcified section at 40x view shows focal areas of odontogenic epithelial islands and fibrous connective with few moderately dense blood vessels and calcification The black arrow shows odontogenic epithelial islands and the white arrow shows calcifications.

The connective tissue associated with another bit was moderately dense, with few blood vessels and focal areas of odontogenic epithelial islands and calcifications (Figure 11).

**Figure 12:**
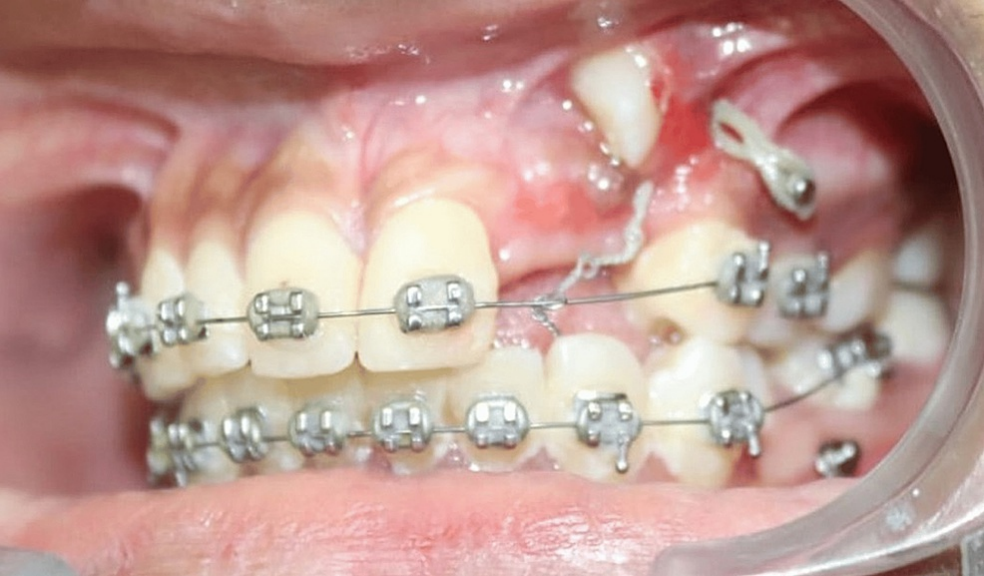
The crown surface of 13 being clinically visible after traction

**Figure 13:**
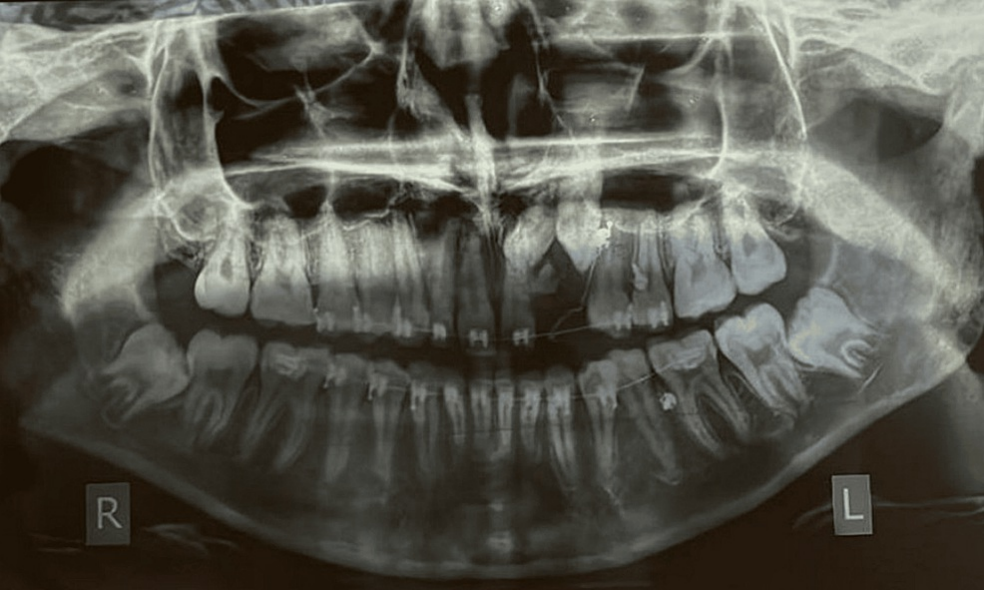
Change in axial inclination of 13 post-traction and favorability for an eruption in the oral cavity

Histopathological features were suggestive of the odontome compound type. Based on the histopathological findings, the final diagnosis is suggested to be a compound odontoma. There were no masses of ghost cells observed in the cystic lumen or in many areas of the fibrous wall, which excludes the possibility of it being a calcifying odontogenic cyst (CCOT) associated with odontoma or a dentigerous cyst associated with odontoma. Therefore, the presence of typical histological features of a compound odontoma supports this conclusion. The post-operative healing was un-inventful (Figures 12, 13).

## Conclusions

In summary, this case sheds light on the intricate interplay between exfoliation-eruptive cycles and odontogenic pathology, underscoring the importance of a multidisciplinary approach for effective management. The innovative use of TADs provided reliable anchorage for orthodontic traction, minimizing the strain on dental units and facilitating successful tooth eruption. This unique case exemplifies the integration of pathology, radiology, and orthodontics in clinical practice, highlighting the collaborative efforts required to address complex dental issues. Moving forward, continued exploration of such interdisciplinary approaches is essential for advancing treatment outcomes and improving patient care in similar challenging cases.
